# An updated pGREEN-based vector suite for cost-effective cloning in plant molecular biology

**DOI:** 10.17912/micropub.biology.000317

**Published:** 2020-10-07

**Authors:** Anna I. Pratt, Jan Knoblauch, Hans-Henning Kunz

**Affiliations:** 1 School of Biological Sciences, Washington State University, PO Box 644236, Pullman, WA 99164-4236, USA; 2 Biozentrum der LMU München Dept. Biologie I - Botanik Großhadernerstr. 2-4 D-82152 Planegg-Martinsried Germany

**Figure 1:**
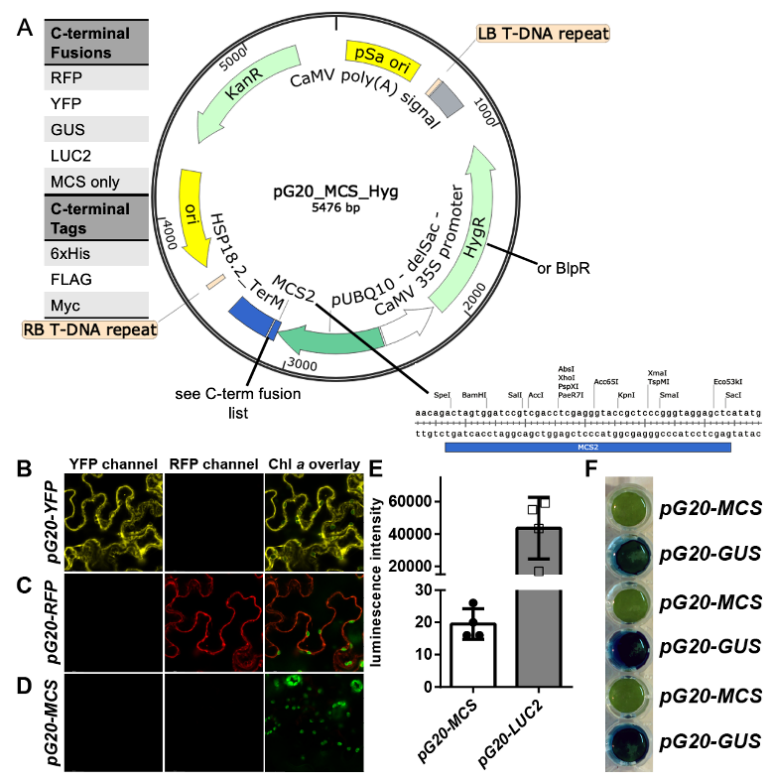
A) Basic map of pG20-toolkit plasmids. B-F) Proof of concept for C-terminal fusion proteins of the pG20 toolkit using transient expression in *Nicotiana benthamiana* leaves. B) Empty YFP and C) empty RFP localized in the cytosol or nucleus. D) As expected, no fluorescence signal was detected when vector pG20-MCS was injected as a control. The same was true in E) the luciferase assay and F) the GUS assay whereas pG20-LUC2 and pG20-GUS respectively gave strong signals.

## Description

Traditional binary shuttle vectors used for creating plant T-DNA insertion mutants can be difficult to work with for several reasons. First, their large size (> 10 kB) and low-copy origin of replication result in low plasmid yields under typical growth conditions. This makes time consuming, and costly, midi or maxi scale plasmid isolation a requirement. Second, accurate sequence information is often not available, rendering vector modification difficult. Finally, cloning techniques that are reliant on restriction enzyme sites prohibit deliberate design of vectors resulting in haphazard configuration of components. Newer cloning techniques, such as Gibson (Gibson *et al.*, 2009) and In-Fusion^TM^ assembly, offer unlimited flexibility but rely on sufficient vector fragment quantity; this can be achieved either by restriction digest or by PCR. However, the large size and low DNA yield of binary shuttle vectors make their use in assembly reactions challenging; this is especially true for large batch DNA library cloning.

Movement of features not essential to cloning helped to alleviate the large size of the Ti plasmid; further modification to ‘helper’ plasmids resulted in the pGREEN system (Hellens *et al.*, 1999). The pGREEN binary vector system offers small plasmid size (5.5 kb) and high copy number of the shuttle vector in *Escherichia coli* (*E. coli).* Although the pGREEN plasmid collection has aged well, many new molecular tools have been introduced. To provide the research community with an updated toolbox, we created a suite of streamlined pGREEN-based vectors that contain several features frequently used in molecular plant science. We designated the plasmids as pG20, short for pGREEN2020, to maintain a reference to the original hallmark work (Hellens *et al.*, 1999). The small size and high DNA yield provide ease of use, while modular construction allows for easy replacement of the promoter and terminator. The inclusion of a multiple cloning site (MCS) with 6‐bp palindromic unique restriction enzymes sites (Waadt *et al.*, 2008) allows full flexibility with restriction-based cloning, either for ligations or to prepare generic low cost Gibson fragments. To lower design costs further, a single primer pair (or one primer within the pair) can be used to yield several different protein tag fusions. Our vectors contain a variety of C-terminal protein-fusion tags; each one comes with two different antibiotic resistance markers allowing for use in many different plant mutant backgrounds. The addition of N-terminal tags could easily be achieved using the ‘tag less’ construct in a Gibson or In-Fusion^TM^ assembly. Plasmids can be obtained from Addgene, the Arabidopsis Biological Resource Center (ABRC), or by contacting our group directly.

A total of 16 constructs were created, using the constitutive AtUBQ10 promoter in combination with the AtHSP18.2 terminator, resulting in strong, tissue-independent expression. The following C-terminal fusions are available: RFP, YFP, GUS, luciferase2, His-tag, FLAG-tag and Myc-tag. In addition, there is a tag-free construct with only the MCS between promoter and terminator (Figure 1A, image generated using Snapgene^TM^ software from GSL Biotech). Each plasmid is available with either hygromycin or glufosinate resistance genes, driven by the CaMV35S promoter. Following confirmation of successful cloning by Sanger sequencing, functionality of the newly designed constructs was demonstrated through transient expression in infiltrated *Nicotiana benthamiana* leaves (Figure 1B-F). Additionally, we have used these constructs to create numerous stable transgenic *Arabidopsis thaliana* lines in a variety of on-going projects.

## Methods

Vectors pGreenII 0179 (Hellens *et al.*, 1999) and pHygII-UT-c-term-Venus (Kunz *et al.*, 2014) were used as the initial templates for creation of Gibson assembly fragments via PCR (Gibson *et al.*, 2009). These products were transformed into electrocompetent TOP10 *E. coli* cells; transformants were selected for on LB media + kanamycin, identified using colony PCR, and verified by Sanger sequencing. Plasmids were isolated using a Qiagen MiniPrep Plasmid kit. The first design yielded pG20_YFP_Hyg, which then served as the parent plasmid for the other hygromycin constructs. Further fragments were either amplified from other vectors: RFP (Waadt *et al.*, 2008), GUS (Nakagawa *et al.*, 2007), LUC2 (Rellán-Álvarez *et al.*, 2015), or synthesized as DNA oligos. The hygromycin constructs were used as templates for the creation of the glufosinate resistance constructs; pFH0032 (Hauser *et al.*, 2013) was used as template to generate the glufosinate resistance gene fragment. The vector suite was then transformed into electrocompetent *Agrobacterium tumefaciens* GV3101 containing the pSOUP helper plasmid (Hellens *et al.*, 1999). Transformants were selected on LB media + rifampicin, gentamycin, tetracycline, and kanamycin, and confirmed by PCR. All bacterial strains were stored at -80°C in LB + 20% glycerol.

Proof of concept studies were carried out by transient expression via infiltration into four week old *Nicotiana benthamiana* leaves. Constructs were assayed three days after infiltration. Fluorescence protein localization studies using a Leica SP8 and GUS stains were performed as described before (Höhner *et al.*, 2019). Luciferase activity was measured using a Tecan M200 plate reader using 4 mm leaf punches floated on 100 ml potassium phosphate buffer 100mM (pH 7.0) containing 2 mM D-Luciferin potassium salt (CAS: 115144-35-9).

## Reagents

**Table:** Table 1: Primers used in this study

***Primer Name***	***Sequence***	***Purpose***	***Origination***
HKP590	tttgttgaaaagtctcaataaagcttcgacgagtcagtaataaacg	Fragment amplification, universal forward – pG20_hyg constructs	This work
HKP591	caaacacatacagcgacttagtttacccgccaatatatcc	Fragment amplification, universal reverse – pG20_hyg constructs	This work
HKP592	tattggcgggtaaactaagtcgctgtatgtgtttgtttgag	Vector amplification, forward – pG20_hyg constructs	This work
HKP593	cgtttattactgactcgtcgaagctttattgagacttttcaacaaagg	Vector amplification, reverse – pG20_hyg constructs	This work
HKP867	gagcagaagctgatctcggaggaagacttgtaagagctcatatgaagatgaagatg	Creation of Myc sequence, forward – pG20_hyg construct	This work
HKP868	ttacaagtcttcctccgagatcagcttctgctccccgggagcggtaccctcg	Creation of Myc sequence, reverse – pG20_hyg construct	This work
HKP869	gactacaaggatgacgatgacaagtaagagctcatatgaagatgaagatg	Creation of FLAG sequence, forward – pG20_hyg construct	This work
HKP870	ttacttgtcatcgtcatccttgtagtccccgggagcggtaccctcg	Creation of FLAG sequence, reverse – pG20_hyg construct	This work
HKP871	catcaccatcaccatcactaagagctcatatgaagatgaagatg	Creation of 6xHis sequence, forward – pG20_hyg construct	This work
HKP872	ttagtgatggtgatggtgatgcccgggagcggtaccctcg	Creation of 6xHis sequence, reverse – pG20_hyg construct	This work
HKP881	ggcatctacttcagatttcggtgacggg	Glufosinate fragment amplification, forward	This work
HKP882	gatcccccctatgagcccagaacgac	Glufosinate fragment amplification, reverse	This work
HKP883	ctgggctcataggggggatcagcttg	Vector amplification, universal forward – pG20_blp constructs	This work
HKP884	cgaaatctgaagtagatgccgaccga	Vector amplification, universal reverse – pG20_blp constructs	This work

**Table:** Table 2: NCBI Accessions, ABRC stock numbers and Addgene IDs

***Construct***	***NCBI Accession***	***ABRC stock number***	***Addgene ID***
pG20_mCherry_Hyg	MT896402	CD3-2834	159701
pG20_mCherry_Blp	MT896403	CD3-2835	159702
pG20_Venus_Hyg	MT896404	CD3-2836	159703
pG20_Venus_Blp	MT896405	CD3-2837	159704
pG20_GUS_Hyg	MT896406	CD3-2838	159705
pG20_GUS_Blp	MT896407	CD3-2839	159706
pG20_LUC2_Hyg	MT896408	CD3-2840	159707
pG20_LUC2_Blp	MT896409	CD3-2841	159708
pG20_MCS_Hyg	MT896410	CD3-2842	159709
pG20_MCS_Blp	MT896411	CD3-2843	159710
pG20_6xHis_Hyg	MT896412	CD3-2844	159711
pG20_6xHis_Blp	MT896413	CD3-2845	159712
pG20_FLAG_Hyg	MT896414	CD3-2846	159713
pG20_FLAG_Blp	MT896415	CD3-2847	159714
pG20_Myc_Hyg	MT896416	CD3-2848	159715
pG20_Myc_Blp	MT896417	CD3-2849	159716
pGreenII 0179	EU048866		
pSoup	EU048870.1		
